# Changes in the hippocampal level of tau but not beta-amyloid may mediate anxiety-like behavior improvement ensuing from exercise in diabetic female rats

**DOI:** 10.1186/s12993-024-00235-0

**Published:** 2024-05-03

**Authors:** Kayvan Khoramipour, Maryam Hossein Rezaei, Amirhossein Moslemizadeh, Mahdieh Sadat Hosseini, Narjes Ebrahimnezhad, Hamideh Bashiri

**Affiliations:** 1 Student Research Committee, School of medicine, Kerman University of Medical Sciences, Kerman, Iran; 2 Department of Exercise Physiology, Faculty of Physical Education, Shahid Bahonar University, Kerman, Iran; 3 Department of Immunology, Tehran University of Medical Sciences, Tehran, Iran; 4 Department of Physiology and Pharmacology, Afzalipour School of Medicine, Kerman University of Medical Sciences, Kerman, Iran; 5 Department of Sports Science, Faculty of Educational Sciences and Psychology, Sistan and Baluchestan University, Zahedan, Iran; 6 Physiology Research Center, Institute of Neuropharmacology, Kerman University of Medical Sciences, Kerman, Iran

**Keywords:** High-intensity interval training, Type 2 diabetes, Cognitive function, Learning, Memory

## Abstract

**Background:**

In the present study, we investigated the effect of high-intensity interval training (HIIT) on cognitive behaviors in female rats with a high-fat diet + streptozotocin (STZ)-induced type 2 diabetes.

**Methods:**

Twenty-four female rats were divided into four groups randomly (*n* = 6): control (C), control + exercise (Co + EX), diabetes mellitus (type 2) (T2D), and diabetes mellitus + exercise (T2D + EX). Diabetes was induced by a two-month high-fat diet and a single dose of STZ (35 mg/kg) in the T2D and T2D + EX groups. The Co + EX and T2D + EX groups performed HIIT for eight weeks (five sessions per week, running on a treadmill at 80–100% of V_Max_, 4–10 intervals). Elevated plus maze (EPM) and open field test (OFT) were used for assessing anxiety-like behaviors, and passive avoidance test (PAT) and Morris water maze (MWM) were applied for evaluating learning and memory. The hippocampal levels of beta-amyloid (Aβ) and Tau were also assessed using Western blot.

**Results:**

An increase in fasting blood glucose (FBG), hippocampal level of Tau, and a decrease in the percentage of open arm time (%OAT) as an index of anxiety-like behavior were seen in the female diabetic rats which could be reversed by HIIT. In addition, T2D led to a significant decrease in rearing and grooming in the OFT. No significant difference among groups was seen for the latency time in the PAT and learning and memory in the MWM.

**Conclusions:**

HIIT could improve anxiety-like behavior at least in part through changes in hippocampal levels of Tau.

**Supplementary Information:**

The online version contains supplementary material available at 10.1186/s12993-024-00235-0.

## Introduction

The escalating prevalence of type 2 diabetes mellitus (T2D) constitutes a pressing apprehension for healthcare systems worldwide. This metabolic disorder affects approximately one in eleven individuals globally and represents the majority, that is, 90%, of all diabetes cases [1, 2]. While acute predictable consequences of T2D such as hyperosmolar hyperglycemia, and diabetic ketoacidosis are common, serious chronic issues such as foot ulcers, retinal damage, chronic kidney disease, nerve damage, and neurocognitive disorders often occur [3]. Based on prior research, diabetes has been associated with alterations in animal behavior [4–6], as evidenced by performance in various behavioral tests, including the open field test (OFT), elevated plus maze (EPM), zero maze, and social interaction tests. In these tests, rats with diabetes exhibited significantly greater levels of anxiogenic activity when compared to their non-diabetic [7]. Increased grooming activity in an unfamiliar environment in rats [8], enhanced retention of passive avoidance training in mice [9, 10], and poor retention of a previously taught avoidance response in a T maze in mice [11] have all been reported.

The incidence of T2DM varies among individuals of distinct racial and ethnic backgrounds [12]. Sex is another determinant of T2D prevalence. Women are more likely than men to develop T2D because of their larger proportion of body fat and more sedentary lifestyle [13, 14]. In addition, mounting evidence suggests sex differences in brain function and behavior [15]. Studies showed that men and women differ in the pattern and prevalence of some neurological disorders [16]. For instance, they have more disease indicators and symptoms [17]. The prevalence rates for cognitive impairments are also higher in women than in men, although this may result from more longevity in women [18].

Since the previous century, along with nutrition and medication, exercise training has been regarded as a cornerstone in managing T2D and its side effects (e.g. behavioral disorders) [19, 20]. Considering that lifestyle modification is recommended before pharmacologic therapy, exercise training is strongly recommended as a safe and easily available method [21, 22]. In the past ten years, several studies [23, 24] have focused on high-intensity interval training (HIIT), which results in physiological adaptations that are comparable to those of chronic moderate-intensity continuous training (MICT) while needs less time. HIIT involves short intervals (30 s to 4 min) of high (more than 80% of maximum heart rate ([HR_max_]) and low-intensity exercise (about 50% of HR_max_) [25]. Our previous study [26] showed that HIIT could decrease T2D-induced anxiety-like behaviors and the levels of tau and beta-amyloid (Aβ) with no effect on learning and memory in male rats. Higher T2D and cognitive impairments prevalence, severe neurological disorder symptoms in women than men, as well as differences in brain function and behavior, have given us a nudge to study the effect of eight weeks of HIIT on anxiety-like behaviors, learning, and memory as well as hippocampal changes in the expression of tau and Aβ in the diabetic female rats.

## Materials and methods

24 female Wistar rats with an average weight of 200 g were purchased from the animal farm of the Kerman University of Medical Sciences. The rats were 8 weeks old. They were kept in polycarbonate cages with a light-dark cycle of 12:12 h and a controlled temperature of 22 1.4 °C on average. The ethics committee of the Kerman University of Medical Sciences approved all animal handling and scarification procedures (Ethic code: IR.KMU.REC.1401.033). The animals were divided into four groups randomly: control (Co), control plus exercise (Co + EX), type 2 diabetes mellitus (T2D), and diabetes plus exercise (T2D + EX). Animals in the T2D and T2D + EX groups were given a high-fat diet for two months and then received a single dose of 35 mg/kg streptozotocin (STZ) intraperitoneally (IP). Three days following the injection, blood glucose was tested using a glucometer. Rats having fasting blood glucose (FBG) levels greater than 300 mg/dl were categorized as diabetic rats for the study [27]. After diabetic induction, an eight-week exercise regimen was started. The open field test (OFT), elevated plus maze (EPM), Morris water maze (MWM), and passive avoidance test (PAT) were completed 24 h after the last training session. Following these tests, all 24 animals (four groups of six rats) were analyzed and hippocampus tissues were removed and the levels of Aβ and Tau were analyzed using a Western blot.

### High-fat diets

The Royan Research Institute provided us with the high-fat diet, which contains the following components: 60% fat (245 g Lard and 25 g Soybean oil), 20% carbohydrate (125 g Lodex 10 and 72.8 g Sucrose), 20% protein (200 g Casein and 3 g Cysteine), 50 g fiber (SOLKA-FLOC), 50 g minerals, 3 g vitamins, and 0.5 g stain [26, 28]. A regular diet has a similar composition to a high-fat diet, except for the percentages of fat (i.e. 10%) and carbohydrates (i.e. 70%) [29, 30].

### High-intensity interval training protocol (HIIT)

The training protocol (K1 protocol) was the same as our previous study on male rats [26, 31–38]. In summary, all animals were accustomed to running on motorized treadmills for five days at a speed of 8 m/min and 0% inclination for 10 min per day. Then they underwent incremental tests until they reached their top speed (V_max_). Every two weeks, the V_max_ of the rats was measured, and the new V_max_ was used to determine relative velocity for the following two weeks. The main protocol consisted of 8 weeks of HIIT performed with 80–100% V_max_, five days a week, the training period was also progressively increased every two weeks, with exercise performed in 12 to 30 min sessions. The interval training was 4–10 with a 2:1 work: rest ratio.

### Elevated plus maze (EPM)

The EPM test was used to measure animal anxiety. The standard procedure described in other investigations was used [39]. The EPM equipment was made up of four wooden arms, two of which were opposed and were open (50 × 10 cm) and the other two were closed (50 × 10 × 40 cm). To prevent rats from falling, the edges of the open arms were provided with a 1-cm plexiglass border. The apparatus was anchored 50 centimeters off the ground. The animal is first placed in the device’s center square to begin the test. A video tracking system (Borj Sanat) captured the animal movements over the course of five minutes while a 100 W light source was placed 120 cm from the apparatus’s center. In an EPM trial, the primary metrics were the time spent in each arm and the number of entrances, which are expressed as percentage open arm times (%OAT), percentage open arm entries (%OAE), and locomotor activity.

### Open field test (OFT)

A 90 by 90 by 30-centimeter translucent plexiglass frame serves as the open field apparatus. Two core and periphery zones make up this gadget. Each rat was placed in the center of the test area, free to wander about. OFT was used to assess anxiety, motor performance, and exploratory behavior in an unfamiliar setting [40]. In a 5-minute experiment, the following variables were measured: distance (cm), time (S), frequency (n) spent in the central zone (min), and the number of rearing and groomings. The Borj Sanat video tracking system was employed.

### **Passive avoidance test (PAT)**

The passive avoidance model is the shuttle box. It is thought that the animal refrains from trying a potentially painful situation because it remembers a previous unpleasant experience [41]. The shuttle box was constructed from two identical plexiglass boxes (20 by 20 by 30 cm). The animal was safest in the light-filled chamber; when he entered the dark one, he was subjected to unpleasant electric shock stimuli. Long-term memory, the acquisition trial, and the habituation phase were all included in the experiment [42]. The animal was gently placed in the white chamber during the habituation phase. The rat was free to migrate to the dark area after five seconds when we raised the barrier separating the white and dark chambers. A disposition took 100 s to complete. The barrier was immediately in place as the animal entered the dark chamber. After 10 s, we returned the animal to its cage without administering any shocks at this stage. After 30 min of the acquisition trial, we brought the animals back into the light-filled space. The shock administered to the rats in the dark chamber after we raised the barrier was the key distinction between the acquisition trial and habituation. Three seconds of a shock (50 Hz, 1 mA) were delivered through the dark chamber’s bottom. After the shock had passed for 20 s, we put it back in its cage. We did it again, this time with a 2-min interval. Repeating this process multiple times helped the animal learn to stay away from the dark side. We examined the animals’ memory retrieval 24 h later. The step-through latency time was measured in seconds and used as a memory recall index when the impediment was removed twenty seconds after the subject was placed inside the light chamber. The longest-lasting animals often need five minutes to complete this portion of the exam. In all of our experiments, an entry was considered when all four paws of the animal were inside a brand-new zone.

### **Morris water maze (MWM)**

The Morris water maze is a trustworthy test for evaluating rats’ spatial learning and memory [43]. This maze is a big, circular tank with a diameter of 140 cm and a depth of 45 cm that is filled with water that is 22–24 °C. The rats jumped out of the water onto a covert platform that was 1.5 cm below the surface and was 15 cm broad and 35 cm high. A variety of visual signals were placed around the equipment on the room’s wall, and their locations stayed constant during the test period. The maze was divided into four quadrants, and the animals were dispersed at random within each of those quadrants. A camera placed above the pool’s center captured footage of the animal’s activity during the experiment. A video-tracking system software was used to measure the spatial learning and memory-related indices, such as the total time required to find the hidden platform (escape latency) (Ethovision, Noldus Information Technology, Netherlands). Each of the three blocks in the training phase, which had a 30-minute break between them, contained four consecutive trials. Rats were randomly thrown into the tank at a predetermined point in each quadrant throughout each trial. The rats were given 60 s to swim to locate the secret platform. The rat was gently placed on the platform and left there for 10 s if it did not find it within 60 s. The overall amount of time needed to find the hidden platform served as a proxy for spatial learning (escape latency). Animals learn to identify the hidden platform during the learning phase, and this is demonstrated by the decrease in their swimming distance and escape latency across successive training blocks. The animal stayed on the platform for 20–30 s when it was discovered before being crated for 20–30 s until the next attempt. By removing the platform in a 60-second probe trial two hours following training trials, the retention of spatial memory was evaluated. As a measure of the retention of spatial memory, the amount of time spent in the target quadrant that formerly housed the platform was counted [44].

### Western blot

Animals were given intraperitoneal injections of ketamine (80 mg/kg) and xylazine (10 mg/kg) to induce anesthesia, and hippocampus tissues were removed 48 h following the last training session. The PBS solution was used to clean the hippocampus. In Ripa buffer solution with protease inhibitor on ice, homogenization was carried out using an ultrasonic homogenizer. The homogenate was centrifuged at 4 °C for 20 min at 13,000 rpm, with the supernatant being stored at -80 °C. The total quantity of Aβ 1–42 and the phosphorylated form of Tau protein were then determined by western blotting. Using the Lowry technique and bovine serum albumin as a reference, the total protein concentration in hippocampus samples was determined. 40 g of protein from each sample was combined with a buffer sample after the concentrations were matched. Then, using an 11% SDS-PAGE gel, electrophoresis was carried out for 75 min. The isolated proteins in the gel were then transferred to PVDF paper. After that, the membrane was left to sit in a 2% block solution overnight at 4 °C. The membrane was then quenched four times, each time being followed by a 5-minute TBST solution wash and a 3-hour incubation with the primary antibodies (SANTA CRUZ BIOTECHNOLOGY, INC. sc-28,365, and sc-21,796) (concentration 1.200) for each of the aforementioned proteins. A secondary antibody (SANTA CRUZ BIOTECHNOLOGY, INC, sc-2357 and sc-516,102) was then applied to the membrane for 1 h at a concentration of 1.1000. The following phase involved recording immune detection using the Chemi Doc XRS + imaging system (Bio-Rad Company, USA) and analyzing the results using ImageJ software and β-Actin served as the control [45].

### Statically analysis

Shapiro-Wilk and Leven’s tests were used to examine the normality and homogeneity of variances, respectively. To compare the Morris water maze’s learning phases, animals’ weight, and FBG a repeated-measures ANOVA and Bonferroni posthoc test was done. Two-way ANOVA was used to evaluate all other data, and it was followed by the Tukey posthoc test. P values under 0.05 were taken into consideration as the standard for statistical significance. Data analysis was done using GraphPad Prism version 8.00 (GraphPad Software, San Diego, USA).

## Results

### The effects of HIIT on the levels of FBG of rats with T2D

**Fig. 1:**
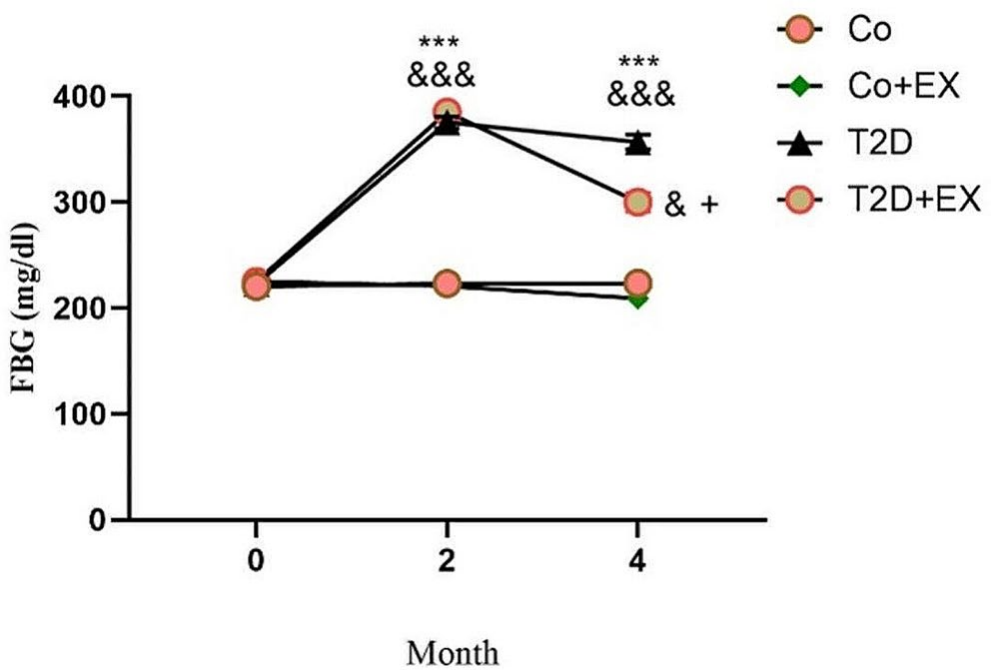
The effects of HIIT on FBG of diabetic rats. The levels of FBG has been illustrated before starting the intervention (month 0), after diabetes induction (month 2), and 48 h after the last training session (month 4). Repeated measures, two-way ANOVA and Tukey post-hoc test was used to evaluate data. Significant differences: ^&^*P* < 0.05 and ^&&&^*P* < 0.001 in comparison with pretest, ***P < 0.001 compared to the control group, ^+^*P* < 0.05 compared to the T2D group. Each bar represents mean ± SEM (*n* = 5 in each group). Co, control; EX, Exercise; T2D, diabetes mellitus (type 2)

FBG was measured to confirm the diabetes induction. Results demonstrated a significant interaction for time and session [F (3, 20) = 3.21, *P* = 0.01]. FBG increased in the female rats after diabetes induction month 2 compared with the pretest (month 0) in T2D and T2D + EX group (*P* < 0.001), with no significant difference between these groups. Increase of FBG continued in month 4 in T2D (*P* < 0.001) and T2D + EX (*P* < 0.05) groups compared to month 0. In addition, in month 4, HIIT could significantly decrease FBG in T2D + EX group in comparison with T2D group (*P* < 0.05). It should be considered that FBG in the T2D group was higher than CO group in month 2 and 4 (*P* < 0.001) (Fig. 1).

### The effects of HIIT on the rat’s weight with T2D

**Fig. 2:**
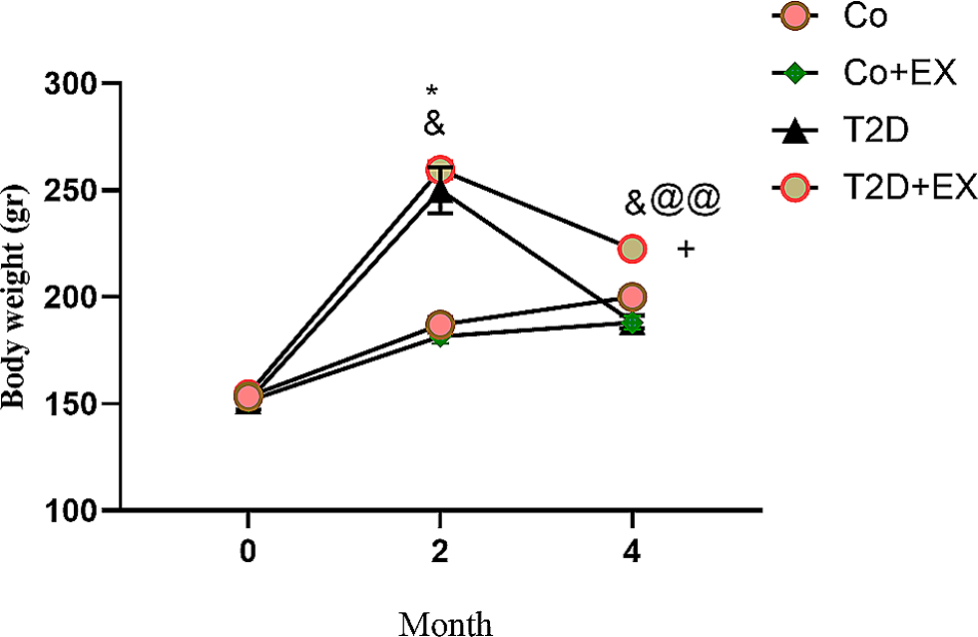
The effects of HIIT on animals’ weight of diabetic rats. The levels of animals’ weight has been illustrated before starting the intervention (month 0), after diabetes induction (month 2), and 48 h after the last training session (month 4). Repeated measures, two-way ANOVA and Tukey post-hoc test was used to evaluate data. Significant differences: ^&^*P* < 0.05 in comparison with pretest, ^@@^*P* < 0.05 compared to month 2, *P < 0.05 compared to the control group, ^+^*P* < 0.05 compared to the T2D group. Each bar represents mean ± SEM (*n* = 5 in each group). Co, control; EX, Exercise; T2D, diabetes mellitus (type 2)

As shown in the Fig. 2, our result revealed a significant interaction for time and session in animals’ weight [F (3, 20) = 2.29, *P* = 0.000]. Animal’s weight significantly increased in T2D (*P* < 0.05) and T2D + EX groups (*P* < 0.001) after diabetes induction in month 2 and 4 compared to pretest. Furthermore, the weight decreased in T2D + EX groups after 4 month compared to month 2 (*P* < 0.01). HIIT could significantly increase weight in month 4 in comparison with T2D group (*P* < 0.05), which may highlight the beneficial effect of HIIT in perserving the muscle weight while reducing the body fat. The weight of rats in the T2D group was higher than CO group in month 2 (*P* < 0.05).

### The effects of HIIT on the %open arm time (%OAT), %open arm entry (%OAE), and locomotor activity of rats with T2D in the elevated plus maze (EPM)

**Fig. 3:**
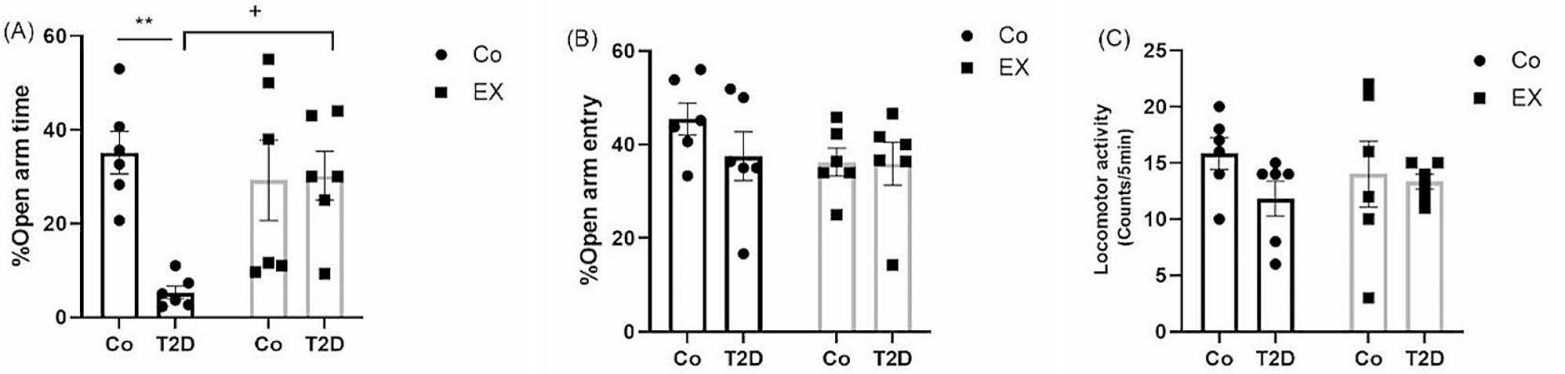
The effects of HIIT on %open arm time (%OAT ((**A**), %open arm entry (%OAE) (**B**), and locomotor activity (**C**) of diabetic rats in the elevated plus maze (EPM). Two-way ANOVA and Tukey posthoc test were used to evaluate data. Significant differences: **P < 0.01 compared to the control group, ^+^*P* < 0.05 compared to the T2D group. Each bar represents mean ± SEM (*n* = 6 in each group). Co, control; EX, Exercise; T2D, diabetes mellitus (type 2)

Figure 3 illustrates the %OAT, %OAE and locomotor activity in the EPM, as a valid task for assessing anxiety-like behaviors. Two-way ANOVA revealed a significant interaction between T2D + EX [F (1, 20) = 7.75, *P* = 0.011], and T2D [F (1, 20) = 6.8, *P* = 0.017], but not for EX [F (1, 20) = 2.92, *P* = 0.10] in the %OAT. Tukey post hoc analysis revealed a considerable difference between the control and T2D groups (*P* < 0.01), indicating anxiety-like behaviors. EX could dramatically increase %OAT in diabetic groups compared to the DM group (*P* < 0.05), suggesting a reversal effect of EX on anxiety-like behavior in the diabetic female rats (Fig. 3A).

As seen in Fig. 3B for %OAE, two-way ANOVA indicated no interaction between T2D + EX [F (1, 20) = 0.86, *P* = 0.37], T2D [F (1, 20) = 0.99, *P* = 0.33], and EX [F (1, 20) = 1.68, *P* = 0.21].

About locomotor activity, two-way ANOVA did not reveal any significant interaction between T2D + EX [F (1, 20) = 0.82, *P* = 0.38], T2D [F (1, 20) = 1.16, *P* = 0.22], and EX [F (1, 20) = 0.008, *P* = 0.93] (Fig. 3C).

### The effects of HIIT on the inner zone distance, inner zone frequency, inner zone time, rearing and grooming of rats with T2D in the open field test (OFT)

**Fig. 4:**
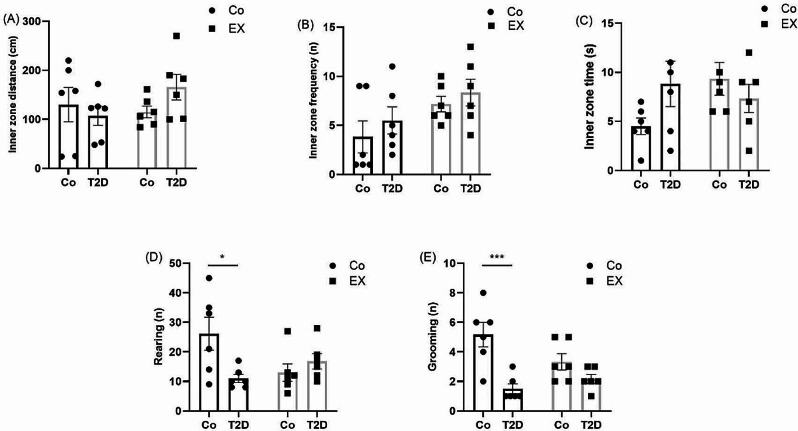
The Effects of HIIT on inner zone distance (**A**), inner zone frequency (**B**), inner zone time (**C**), rearing (**D**), and grooming (**E**) of diabetic rats in the open field test (OFT). Two-way ANOVA and Tukey posthoc test were used to evaluate data. Significant differences: *P < 0.05 and ***P < 0.001 compared to the control group. Each bar represents mean ± SEM (*n* = 6 in each group). Co, control; EX, Exercise; T2D, diabetes mellitus (type 2)

Figure 4A demonstrates the effects of HIIT on the inner zone distance of OFT in female rats. Two-way ANOVA indicated no interaction between T2D + EX [F (1, 20) = 2.21, *P* = 0.16], T2D [F (1, 20) = 0.31, *P* = 0.58], and EX [F (1, 20) = 0.78, *P* = 0.39].

As seen in Fig. 4B for inner zone frequency, two-way ANOVA dramatically indicated an interaction for EX [F (1, 20) = 5.37, *P* = 0.03], but not between T2D + EX [F (1, 20) = 0.03, *P* = 0.85], and T2D [F (1, 20) = 1.13, *P* = 0.30]. Tukey post hoc analysis revealed no significant differences among experimental groups.

About inner zone time, two-way ANOVA did not reveal any significant interaction between T2D + EX [F (1, 20) = 3.68, *P* = 0.07], T2D [F (1, 20) = 0.50, *P* = 0.49], and EX [F (1, 20) = 1.10, *P* = 0.32] (Fig. 4C).

For rearing, as shown in Fig. 4D, two-way ANOVA revealed a significant interaction between T2D + EX [F (1, 20) = 7.33, *P* = 0.01], but not for T2D [F (1, 20) = 2.61, *P* = 0.12], and EX [F (1, 20) = 1.09, *P* = 0.31]. Tukey post hoc analysis revealed a significant reduction in the number of rearing of the T2D group (*P* < 0.05) compared to the control group.

Fig. 4E illustrates the number of grooming in the experimental groups. Two-way ANOVA revealed a significant interaction between T2D + EX [F (1, 20) = 5.14, *P* = 0.03], T2D [F (1, 20) = 19.20, *P* = 0.0003], but not for EX [F (1, 20) = 1.12, *P* = 0.3]. This index in the diabetic rats was dramatically less than in the control group (*P* < 0.001)

### The effects of HIIT on step-through latency of rats with T2D in the passive avoidance test (PAT)

**Fig. 5:**
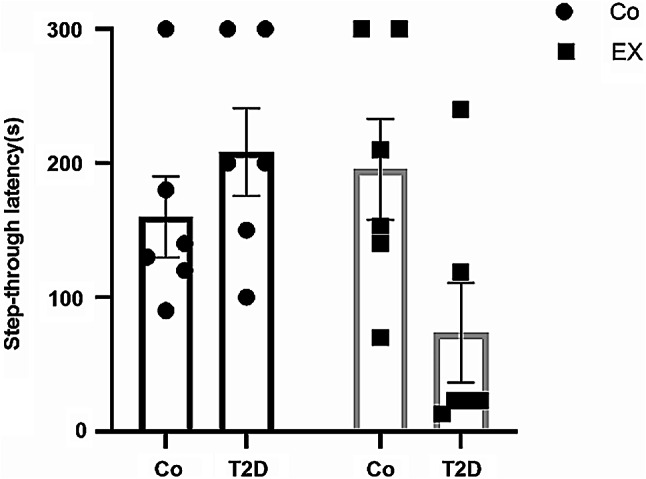
The Effects of HIIT on step-through latency of diabetic rats in the passive avoidance test (PAT). Two-way ANOVA and Tukey post hoc test were used to evaluate data. Each bar represents mean ± SEM (*n* = 6 in each group). Co, control; EX, Exercise; T2D, diabetes mellitus (type 2)

Figure 5 provides the results obtained from avoidance memory in the PAT. Two-way ANOVA revealed a significant difference between T2D + EX [F (1, 20) = 6.06, *P* = 0.02], but not for T2D [F (1, 20) = 1.13, *P* = 0.29], and EX [F (1, 20) = 2.06, *P* = 0.17], suggesting no memory impairment in diabetic rats.

### The effects of HIIT on spatial learning and memory of rats with T2D in the Morris water maze (MWM)

**Fig. 6:**
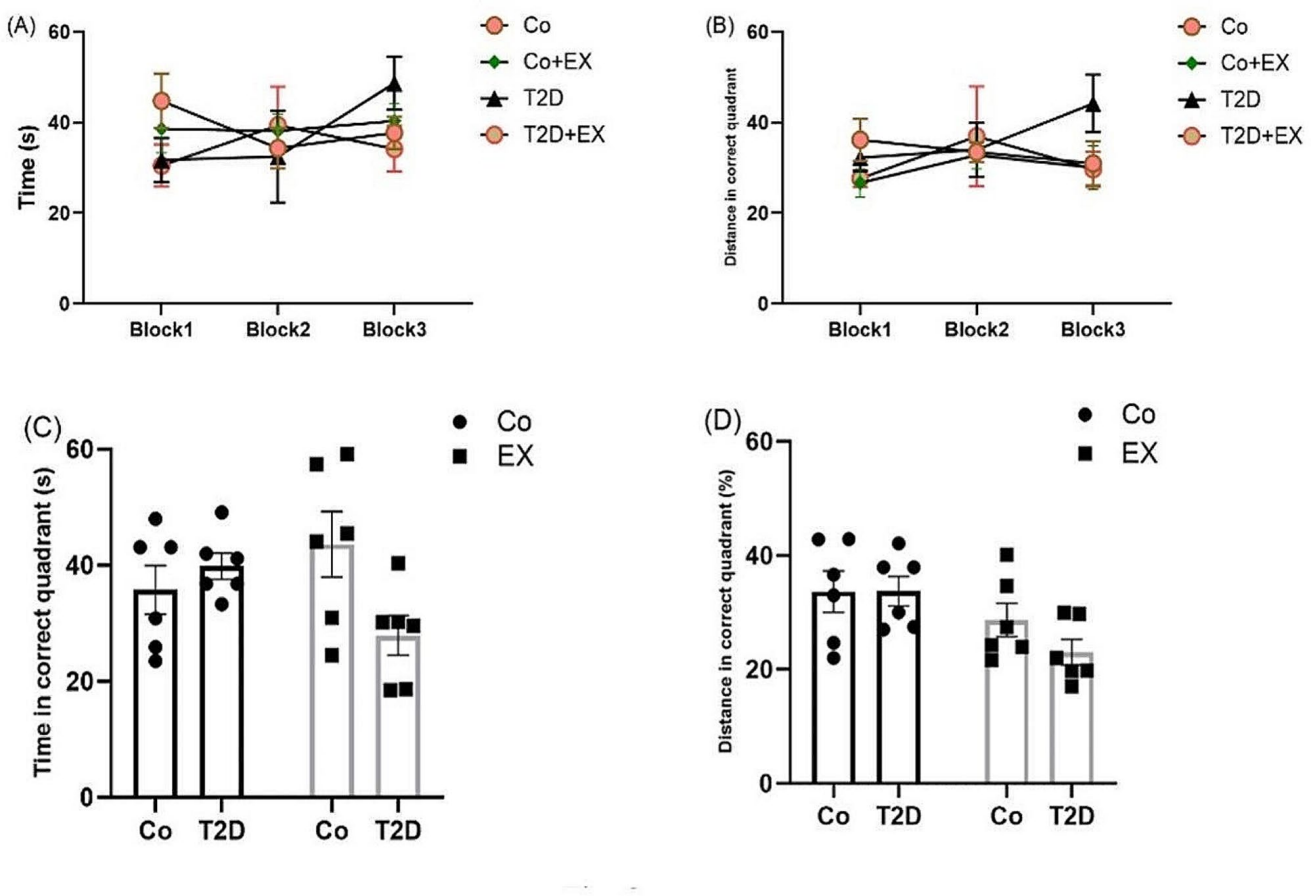
The Effects of HIIT on time spent in each block [learning] (**A**), distance in the correct quadrant [learning] (**B**), time spent in the target quadrant [memory] (**C**), and distance in the correct quadrant [memory] (**D**) of diabetic rats in the Morris water maze (MWM). Two-way ANOVA, repeated measures, and Tukey posthoc test was used to evaluate data. Each bar represents mean ± SEM (*n* = 6 in each group). Co, control; EX, Exercise; T2D, diabetes mellitus (type 2)

Figure 6 illustrates spatial learning and memory in the MWM. Our analysis using repeated measures, and two-way ANOVA did not show a significant difference in time and distance to find the platform among groups, indicating no defect in spatial learning for the diabetic rats with and without HIIT (Fig. 6[A] and [B]).

In the recall probe, two-way ANOVA revealed a significant difference between T2D + EX [F (1, 20) = 5.93, *P* = 0.02], but not between T2D [F (1, 20) = 2.02, *P* = 0.17], and EX [F (1, 20) = 0.25, *P* = 0.62] for time spent in the target quadrant, indicating no defect in spatial memory for the diabetic rats with and without HIIT (Fig. 6C).

About distance in the correct quadrant, two-way ANOVA revealed a significant difference between EX [F (1, 20) = 7.33, *P* = 0.013], but not for T2D + EX [F (1, 20) = 0.97, *P* = 0.33], and T2D [F (1, 20) = 0.92, *P* = 0.34]. suggesting no memory impairment in diabetic rats (Fig. 6D).

### The effects of HIIT on tau and Aß levels in the hippocampus of rats with T2D

**Fig. 7:**
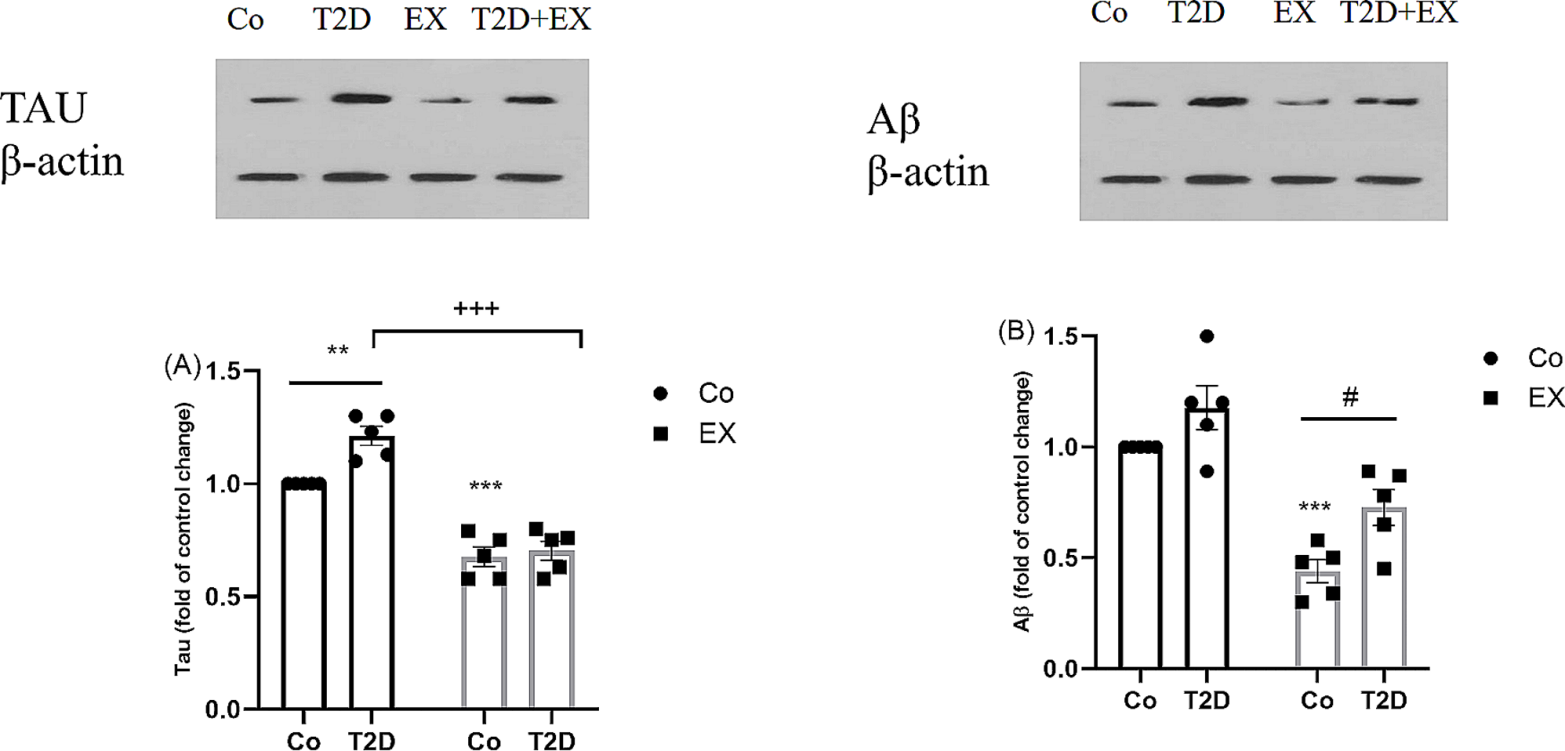
The Effects of HIIT on Tau (**A**) and Aß (**B**) protein levels in the Hippocampus of diabetic rats. Two-way ANOVA and Tukey posthoc test were used to evaluate data. Significant differences: **P < 0.01 and ***P < 0.001 compared to the control group; ^+++^*P* < 0.001 compared to the T2D group; #*P* < 0.05 compared to the Co + EX group. Each bar represents mean ± SEM (*n* = 5 in each group). Co, control; EX, Exercise; T2D, diabetes mellitus (type 2)

Figure 7 (A) and (B) provide the results obtained from the expression level of Tau and Aß in the hippocampus. Two-way ANOVA revealed a significant interaction between T2D + EX [F (1, 20) = 6.31, *P* = 0.02], T2D [F (1, 20) = 10.73, *P* = 0.004], and EX [F (1, 20) = 129, *P* = 0.0001] for the Tau level. Tukey post hoc analysis revealed a considerable increase in the T2D group compared to the control group (*P* < 0.01). In addition, a significant decrease was seen in the Co + EX compared to the control group (*P* < 0.001). The T2D + EX group could dramatically decrease the level of Tau protein in comparison with the T2D group (*P* < 0.001) (Fig. 7A).

As seen in Fig. 7B, two-way ANOVA indicated that there is a significant interaction between T2D [F (1, 20) = 11., *P* = 0.004], and EX [F (1, 20) = 53.61, *P* = 0.0001], but not between T2D + EX [F (1, 20) = 0.63, *P* = 0.43] in the Aß level. Tukey post hoc analysis revealed a significant decrease in the Co + EX compared to the control group (*P* < 0.001). The T2D + EX group could increase the level of Aß protein in comparison with the Co + EX group (*P* < 0.05).

## Discussion

Our data showed that HIIT attenuated anxiety-like behaviors, and levels of Tau in the hippocampus of diabetic female rats. MWM and PAT results did not show a significant difference among the groups, but EPM and OFT data indicated that T2D induced anxiety-like behaviors. This destructive effect of T2D leads to the production and accumulation of Tau which are considered the important risk factors for anxiety-like behavior in diabetic female rats.

The first effective step in managing and preventing diabetes and related memory disorders and anxiety comorbidity is to have a thorough understanding of the underlying neurobiological mechanisms [46, 47]. In this line, we observed that diabetes increased FBG in the female diabetic rats, and caused anxiety-like behaviors. Several studies have reported the relationship between anxiety disorder and T2D [48–51]. For instance, the results obtained from one study showed that diabetic female rats, because of having a larger proportion of body fat, exhibit more anxiety-like behavior compared to male rats [52]. Sivanathan et al. [53] reported that a high-fat diet increased anxiety-like behavior and decreased transcription of glucocorticoid signaling genes in the hippocampus of female rats. Despite the destructive effect of diabetes, our results showed that HIIT could reduce FBG in diabetic rats. Our results are in agreement with several studies [51, 54–56]. Moreover, Previous studies demonstrated high-fat diet and T2D induction increased body weight, and exercise training decreased it in diabetic rats, and as a result exercise could attenuate behavioral disorders [26, 50, 54–57]. In agreement with above-mentioned investigations, T2D could dramatically increase the body weight in our study. The T2D group showed a greater reduction in weight compared to the T2D + EX group which may indicate the beneficial effect of HIIT in preserving the muscle weight while reducing the body fat. Consistent with this, food intake was higher in T2D and T2D + EX compared to other groups. Surprisingly, weight loss in the T2D group was seen in month 4. Diabetes disrupts glucose absorption, which may explain why despite increased food intake, body weight decreased in the T2D group.

In the current study, OFT and EPM were used to assess anxiety-like behaviors. OFT showed that diabetes could decrease rearing and grooming in the female rats with no significant difference in inner zone time, frequency, and distance. In agreement with our results, Caliskan et al. [57] reported that there were not any significant differences in inner zone time and frequency between diabetic and non-diabetic rats. Another important finding in the current study was that HIIT did not have a significant effect on OFT indices. In contrast with our data, several studies [46, 57, 58] showed that exercise training could increase inner zone time, frequency, distance, and locomotor activity baseline in male and female rodents. Furthermore, some investigations displayed that exercise training can increase rearing and grooming which leads to a decrease in anxiety-like behaviors and depression [59, 60]. In addition, many studies [60–63] assessed the effect of sex differences in OFT and they observed anxiety-like behavior increased [60, 62, 63], decreased [64], and do not have a significant difference [61] in female rats compared to males. Inconsistent results may be due to the type of exercise (i.e. moderate intensity in the other studies versus HIIT in our study), and also the gender of the rats.

In addition, in the EPM, we found a similar result with previous studies [46, 57, 65, 66] in which diabetes decreased %OAT, suggesting anxiety-like behaviors in the T2D group. HIIT could reduce anxiety disorder which was proved through increased %OAT in the T2D + EX group. Also, we previously assessed the effect of HIIT on the EPM indices in diabetic male rats [26], and our results showed that both %OAT and %OAE increased in the T2D + EX group, indicating anxiolytic behavior. Naturally-cycling and ovarian hormones in female rats [15, 67] may explain this little inconsistency. Interestingly, female rats exhibit a reduction in generalized anxiety at the onset of estrus, when estradiol has returned to baseline and progesterone is declining. This suggests that the natural fluctuations in estradiol and progesterone across the estrous cycle may promote changes in anxiety [68]. Elucidating the exact mechanism underlying sex differences in the diabetic requires further studies.

We also evaluated passive avoidance memory, spatial learning, and memory with PAT and MWM, respectively. In contrast to several studies [69–71], our results did not show any significant difference among all groups in STL (Step-Through Latency), time in the correct quadrant, and distance in the correct quadrant in the above-mentioned tests. In agreement with our data, Sadati et al. [72] demonstrated that four weeks of treadmill exercise (30 min during 2 consecutive days and 15 m/min speed) had no obvious effect on learning and memory in the MWM in female rats. Mohammadi et al. [73] showed that treadmill exercise (5 days/week, 10 m/min, 30 min/day for the first two weeks, and 15 m/min, 30 min/day for the last two weeks) did not change spatial learning in the ovariectomized diabetic rats. Although the secretion of estrogen could describe contrasting effects between genders on memory [15], we showed a similar effect in male rats [26] and we assume these changes merely reflect baseline differences in activity. However, more studies are needed.

Our data also showed that diabetic rats displayed higher hippocampal Tau accumulation levels than non-diabetics. We also found that HIIT could significantly reduce TAU levels in the T2D + EX group. Previous studies [74, 75] have shown that Aβ and Tau accumulation plays a vital role in the pathogenesis of cognitive function and anxiety-like disorders in diabetic rats. In line with part of our findings, several studies [74–78] have shown that exercise could significantly decrease Tau accumulation levels and improve memory and anxiety-like behaviors. However, the result of the current as well as our previous study on the male animals showed that HIIT-induced hippocampal TAU changes were associated with improvement in anxiety-like behavior but not memory in rats with T2D [26]. Smith et al. [79], reported that TAU accumulation was higher in female than male subjects with neurological impairments which may highlight the vital role of TAU in the pathogenesis of neurological impairments especially in females. Failing to see the improvement in memory could be in part explained by the type of exercise (low and moderate-intensity training, running wheel, and swimming), the dose of STZ, the method of diabetic induction, and the species of animal. Prospective studies focusing on the evaluation of neurobiological mechanisms underlying behavioral functions in different genders may shed light on this topic.

## Conclusion

Our findings demonstrated that a high-fat diet and STZ injection in the female rats caused T2D, which increased anxiety-like behaviors without any effect on learning and memory. On the other hand, HIIT as a safe and effective intervention, at least in part through the reduction of Tau, could attenuate anxiety-like behaviors. Additional studies are needed to determine the exact effect of gender on signaling pathways of cognitive behaviors during HIIT intervention.

### Electronic supplementary material

Below is the link to the electronic supplementary material.

Supplementary Material 1

## Data Availability

The datasets used and/or analyzed during the current study are available from the corresponding author upon reasonable request.

## Abbreviations

HIIT: High-intensity interval training
STZ: Streptozotocin
Co: Control
Co + EX: Control + exercise
T2D: Diabetes mellitus (type 2)
T2D + EX: Diabetes mellitus + exercise
EPM: Elevated plus maze
OFT: Open field test
PAT: Passive avoidance test
MWM: Morris water maze
Aβ: Beta-amyloid
%OAT: Percentage Open arm time
