# Supplementary figures and images for: Changes in the hippocampal level of tau but not beta-amyloid may mediate anxiety-like behavior improvement ensuing from exercise in diabetic female rats
Source: Behav Brain Funct. 2024 May 3;20:9. doi: 10.1186/s12993-024-00235-0 (PMC11067136; doi:10.1186/s12993-024-00235-0)

**AB**


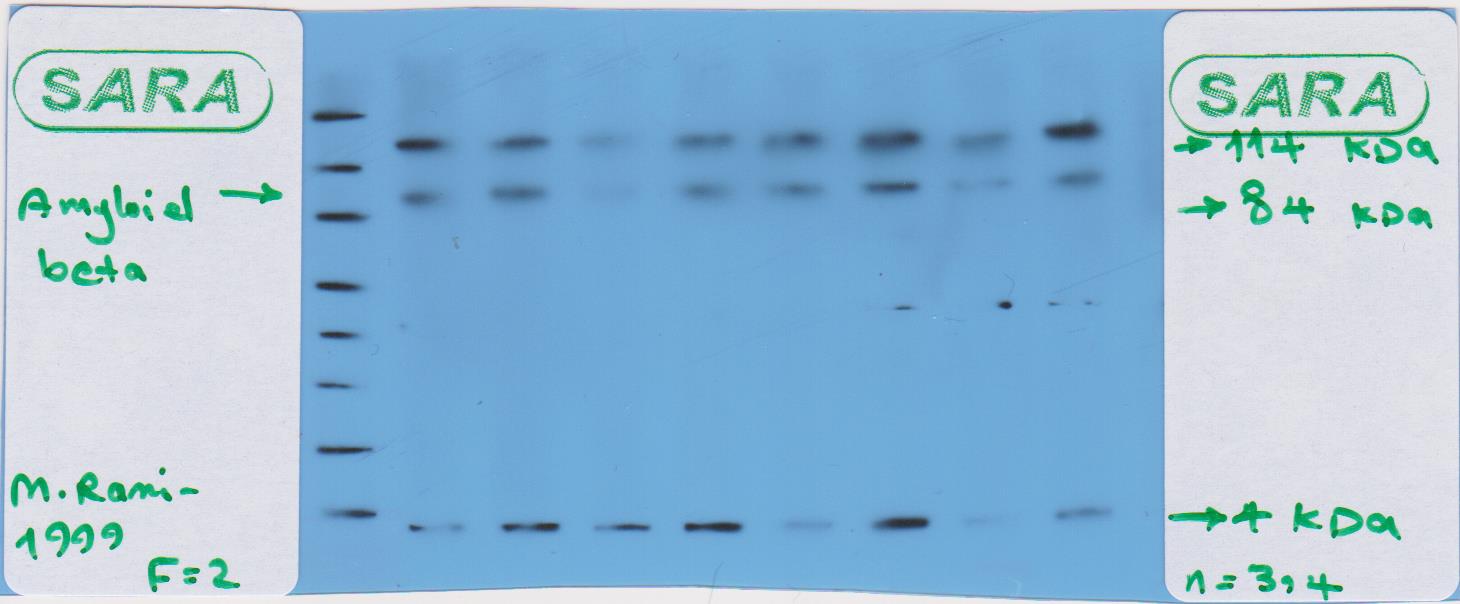


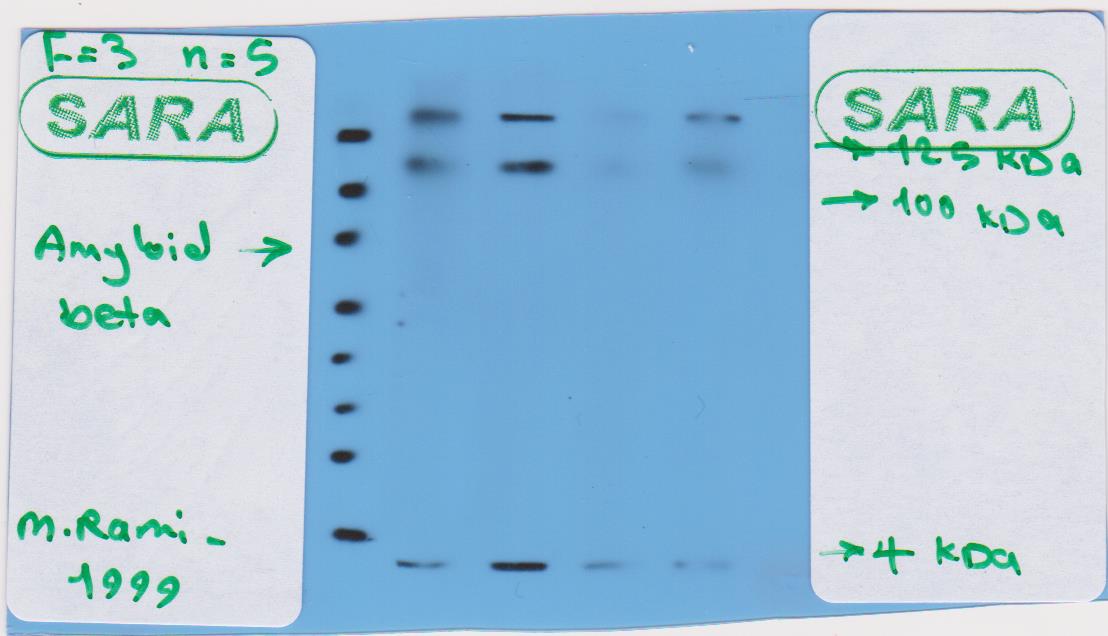


**TAU**


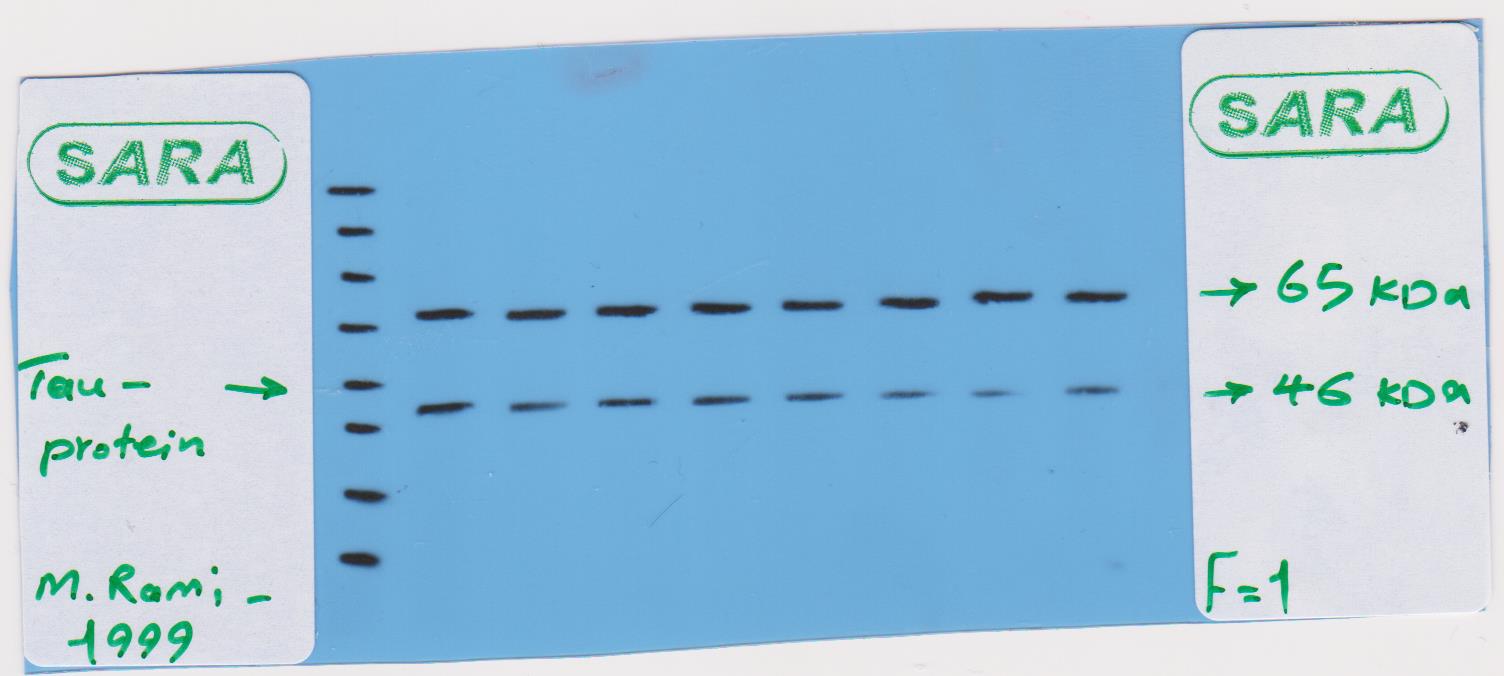


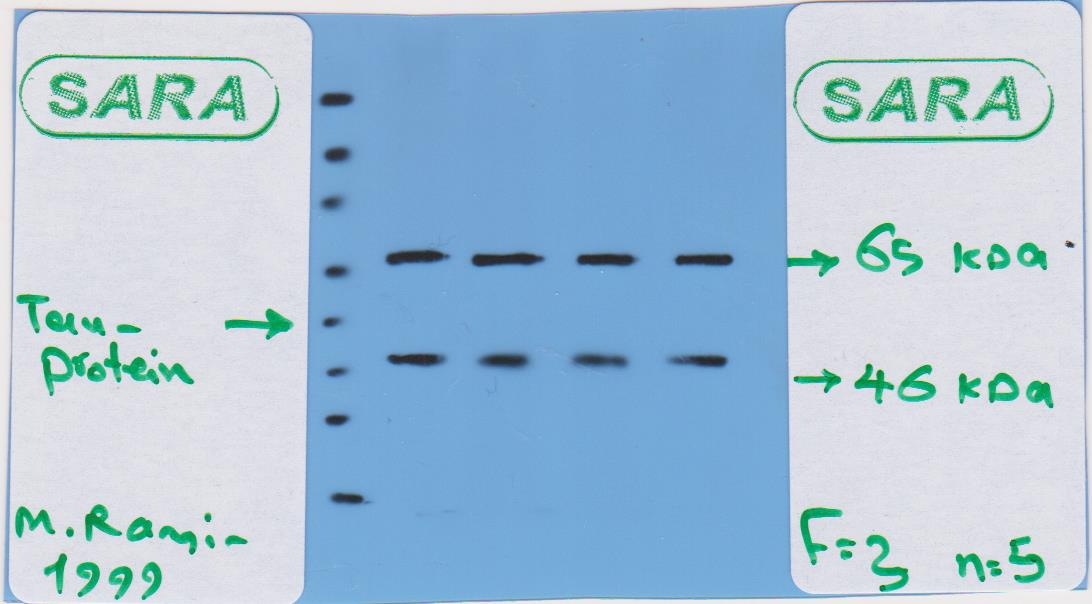

Supplement: Supplementary file 1 — Supplementary Material 1 [file 12993_2024_235_MOESM1_ESM.docx]
